# Poly-3-hydroxybutyrate-co-3-hydroxyvalerate(PHBV)-Polyethylene glycol 20k(PEG20k) as a promising delivery system for PT2399 in the treatment of disc degeneration

**DOI:** 10.1186/s13036-024-00407-6

**Published:** 2024-01-22

**Authors:** Zhencong Li, Weilin Zhang, Shengbang Huang, Zhiwen Dai, Jinguo Liang, Qiulan Qiu, Siyuan Chen, Weixiong Guo, Zhongwei Wang, Jinsong Wei

**Affiliations:** 1 Department of Spinal Degeneration and Deformity Surgery, Affiliated Hospital of Guangdong Medical University, Zhanjiang, 524001 China; 2 School of Public Health, Guangdong Medical University, Dongguan, 523808 China

**Keywords:** PHBV, Nucleus pulposus, HIF-2α, PT2399, Anoxic environment

## Abstract

Disc degeneration often leads to a highly prevalent symptom known as low back pain. Healthy nucleus pulposus tissue exhibited a hypoxic environment devoid of blood vessels, while degenerated nucleus pulposus experienced hypoxic deterioration and the formation of new blood vessels. In this study, the expression of important genes like HIF-2α was found to vary between normal and degenerated nucleus pulposus cells when compared to the hypoxic surroundings. The aim of this study was to examine how HIF-2α is controlled in nucleus pulposus cells under hypoxic conditions and its role in angiogenic mechanisms. To assess the impact of gradual inhibition of HIF-2α on disc degeneration, we utilized PHBV-based synthetic materials loaded with inhibitors of HIF-2α. Specifically, we employed LPS and PT2399 loaded PHBV-PEG20k (PP20) to intervene with human nucleus pulposus cells. Additionally, we treated APD rat models with PT2399 loaded PP20 to evaluate its effects. The expression levels of target markers in nucleus pulposus cells were detected using PCR, WB, and immunofluorescence. Additionally, the effect of drugs on disc degeneration was identified through HE staining. The findings indicated that HIF-2α, CAIX, PPP1R15A, VEGFA, and EGLN3 could potentially serve as new indicators of disc degeneration. Additionally, HIF-2α might contribute to the progression of disc degeneration through involvement in angiogenesis and the regulation of hypoxia. Furthermore, the utilization of PT2399 loaded PHBV-PEG20k (PP20) could potentially offer a fresh alternative for treating disc degeneration.

**Supplementary Information:**

The online version contains supplementary material available at 10.1186/s13036-024-00407-6.

## Introduction

Backache is an extremely prevalent indication and currently ranks as the primary factor behind global incapacitation [1]. Intervertebral disc (IVD) degeneration (IDD) is a significant contributor to both chronic disability and the prevalence of low back pain [2]. Degeneration of the intervertebral disc, which consists of the nucleus pulposus, annulus fibrosus, and endplate, results in an atypical distribution of stress within the spine. The disc may become narrower due to biomechanical compensation, leading to calcification of the facet joint and narrowing of the foraminal space. This can result in nerve entrapment and irritation, causing chronic low back pain and a decrease in quality of life [3]. Because of this, research on intervertebral disc degeneration has become a hot topic. Prior research has identified inflammation, trauma, oxidative stress, aging, and apoptosis as factors contributing to disc degeneration; however, the exact mechanism behind this degeneration is still not fully understood [4].

Hypoxia damage is an important link in the pathological mechanism of the human body.The nucleus pulposus tissue in a healthy adult human has a structure without blood vessels and relies on anaerobic metabolism in a hypoxic environment [5, 6]. However, in the degenerative state of the intervertebral disc, the nucleus pulposus exhibits angiogenesis [7, 8]. Earlier research has indicated that the equilibrium of HIF-1α and HIF-2α is responsible for maintaining the oxygen-deprived environment of nucleus pulposus cells [9]. Additionally, the regulation of HIF-1α plays a crucial role in the glycolysis and mitochondrial metabolism of nucleus pulposus cells [10]. On the other hand, eliminating HIF-2α has been found to decrease fibrosis [11], although the exact mechanism of its regulation is still unknown.Further exploration into the regulation of oxygen levels in the nucleus pulposus could potentially unveil novel avenues for future treatments targeting degenerative lumbar disc disease.Hence, it is crucial to acquire understanding of the regulation of HIF-2α and its associated mechanisms in nucleus pulposus cells while experiencing degeneration.

HIF-2α is recognized to have connections with angiogenesis and various other processes in different tissues [12–15]. On the other hand, PT2399 is a specific inhibitor of HIF-2α that hinders and separates it [16]. By taking into account the traits of non-sustained release and inadequate stability in individual drug components, employing biological loading materials can potentially attain the attributes of slow-release therapy. For numerous years [17, 18], PHBV (poly-3-hydroxybutyrate-co-3-hydroxyvalerate), an organic polymer compound, has been employed in orthopedics and various medical domains due to its piezoelectric characteristics. Due to its remarkable durability and flexibility, it is a perfect substance for various medical uses, such as transporting medications, packaging medical supplies, stitching wounds, and creating cardiovascular stents. Despite this, the material has poor hydrophilicity, resulting in poor tissue compatibility, which limits its clinical application. Nevertheless, the drawback has been significantly altered by the introduction of the novel substance PP20, also known as PHBV-PEG20k (polyethylene glycol 20k).

Chronic and structural changes define disc herniation, a degenerative condition. Addressing the degeneration of nucleus pulposus tissue is an essential component in the management of disc degeneration. To investigate the biological processes linked to this in nucleus pulposus cells, we discovered hypoxia-related genes that were expressed differently in normal and degenerated nucleus pulposus tissues in a database. Then, we treated nucleus pulposus cells with lipopolysaccharide (LPS) to induce degeneration and loaded them with PT2399 and PP20. Finally, we examined the impact of PT2399 loaded PP20 on disc degeneration using an APD rats model. Measuring the target index, HIF-2α, CAIX, PPP1R15A, VEGFA, and EGLN3 could potentially serve as new indicators for disc degeneration. Additionally, HIF-2α might contribute to the progression of disc degeneration through its involvement in angiogenesis and regulation of hypoxia. The objective of this study was to investigate the possible influence of enhanced delivery methods utilizing optimized PHBV carriers in the treatment of IDD. The results of our study enhance the comprehension of the process of disc degeneration and establish a theoretical foundation for future treatments of this condition.

## Results

### Gene expression disparities between human normal and degenerated nucleus pulposus cells are unveiled through single cell sequencing

**Fig. 1:**
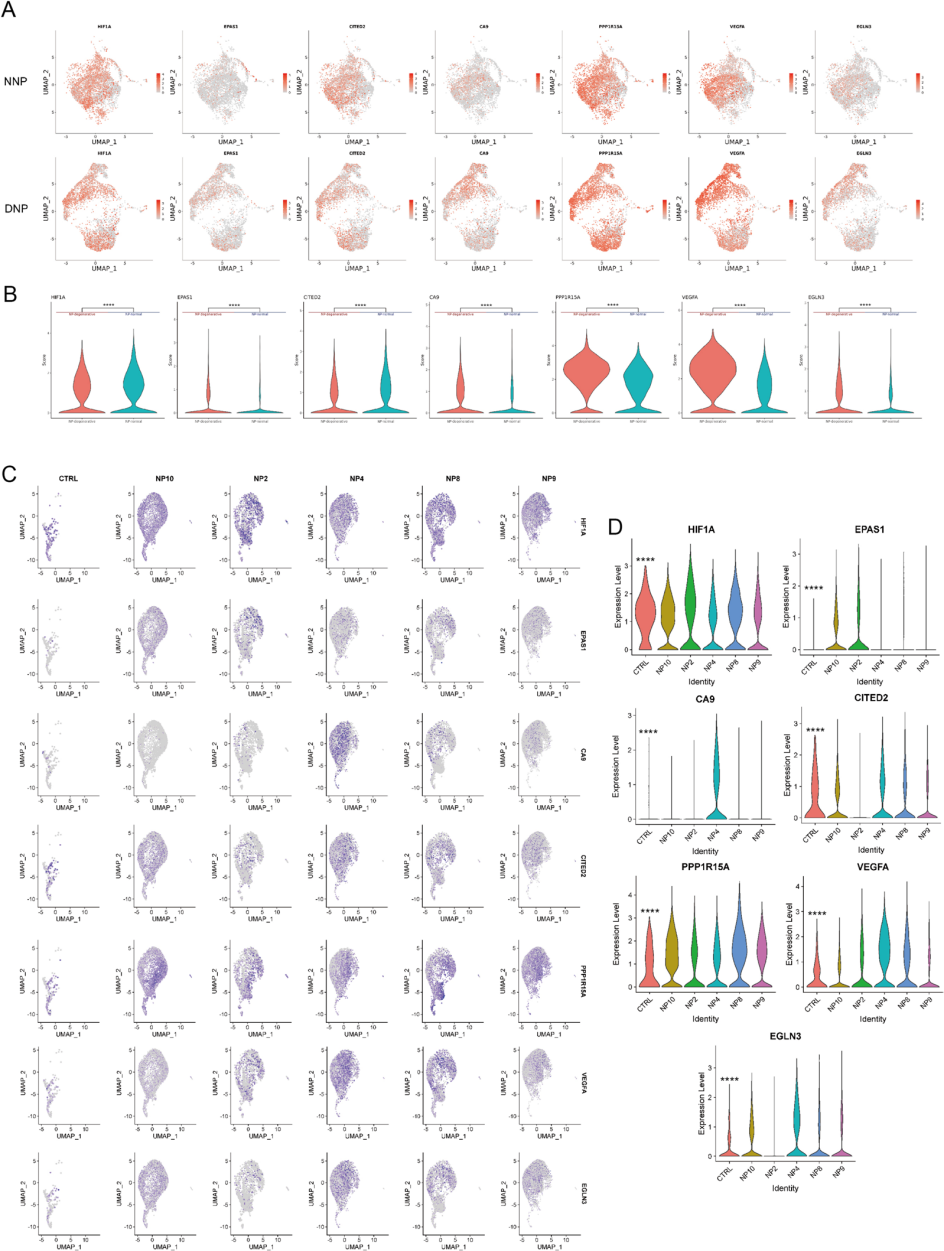
The UMAP plots display the distribution of gene expression in human normal and degenerated nucleus pulposus samples from the GSE205535 database. Violin plots were utilized to compare the gene expression of various genes in samples of both healthy and degenerated nucleus pulposus obtained from the GSE205535 database. UMAP plots display the distribution of gene expression in samples of human normal and degenerated nucleus pulposus from the CNP0002664 database. Comparisons were made between the expression of various genes in samples of human normal and degenerated nucleus pulposus from the CNP0002664 database, using violin diagrams. (****p < 0.0001)

**Fig. 2:**
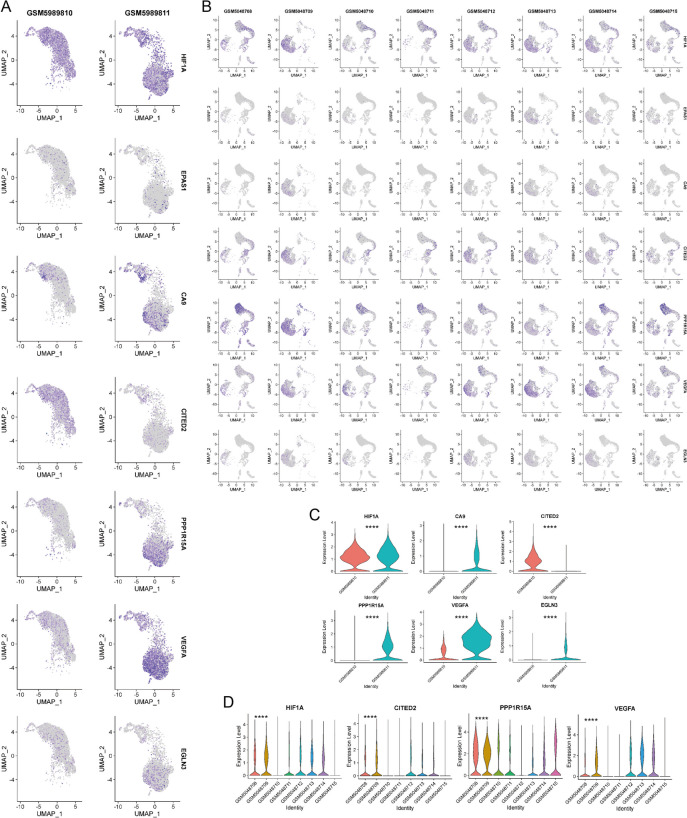
The GSE199866 database displays UMAP plots illustrating the distribution of gene expression in human normal and degenerated nucleus pulposus samples. (B) UMAP plots showed the distribution of expression of different genes in nucleus pulposus samples with different grades of degeneration in humans in the GSE165722 database. Violin plots were utilized to compare the gene expression of various genes in samples of human normal and degenerated nucleus pulposus obtained from the GSE199866 database. In the GSE165722 database, violin plots were used to compare the gene expression of various genes in human nucleus pulposus samples exhibiting varying levels of degeneration. (****p < 0.0001)

The comparison of differentially expressed genes in human normal and degenerated nucleus pulposus cells was conducted using the Gene Expression Omnibus (GEO) database and the China National GeneBank DataBase (CNGBdb) database. This analysis revealed the identification of certain genes associated with hypoxia and angiogenesis (Figs. 1, and 2). In these results, which showed differences in gene expression, there were statistically significant differences between the human normal nucleus pulposus and degenerated nucleus pulposus groups.

Single-cell sequencing in GSE205535 [19] identified differential cellular profiles and expressed gene variations between a healthy human nucleus pulposus tissue and a degenerated human nucleus pulposus tissue. During the examination of this database, it was discovered that HIF-1α and CITED2 exhibited increased levels in human normal nucleus pulposus cells (NNP). Conversely, HIF-2α (EPAS1), CA9, PPP1R15A, VEGFA, and EGLN3 demonstrated significant expression in human degenerated nucleus pulposus cells (DNP) (Fig. 1B). Analysis of dimensionality reduction showed that the distribution of gene expression varied among various types of nucleus pulposus cells (Fig. 1A).

Single cell sequencing was performed in 5 degenerated nucleus pulposus samples (NP2, NP4, NP8, NP9, NP10) and 1 control normal nucleus pulposus sample (CTRL) in a study on the CNP0002664 database [20]. We analyzed it and found that its gene expression distribution was also regular. The gene expression level violin plot revealed that the normal nucleus pulposus cells (CTRL) exhibited higher expression levels of HIF-1α and CITED2 compared to degenerated nucleus pulposus cells, along with a greater number of cells expressing these genes.Similarly, HIF-2α (EPAS1), CA9, PPP1R15A, VEGFA, and EGLN3 were highly expressed to varying degrees in human degenerative nucleus pulposus (Fig. 1 C,D).

In the study GSE199866 [21], four different types of tissue samples were analyzed, which included healthy annulus fibrosus tissue, degenerated annulus fibrosus tissue, healthy nucleus pulposus tissue, and degenerated nucleus pulposus tissue from humans. We performed dimensionality reduction analysis of different gene expression distributions in human healthy nucleus pulposus samples (NPH, GSM5989810) and human degenerated nucleus pulposus samples (NPD, GSM5989811) and violin diagram to show gene expression in different samples (Fig. 2 A,C).HIF-1α and CITED2 showed higher expression levels in GSM5989810 (NPH), while CA9, EGLN3, PPP1R15A, and VEGFA were more highly expressed in GSM5989811 (NPD), with VEGFA varying to a greater extent in the two samples. The database of this study did not show any significant difference in HIF-2α expression, and only the distribution of dimensionality reduction was presented.

In the study GSE165722 [22], single cell sequencing analysis was conducted on a combined 8 samples of nucleus pulposus tissues with varying levels of degeneration. These samples included GSM5048708, GSM5048709 (Grade I), GSM5048710, GSM5048711 (Grade II), GSM5048712, GSM5048713 (Grade III), GSM5048714, and GSM5048715 (Grade IV). Additionally, the analysis of hypoxia and angiogenesis associated genes in nucleus pulposus cells with varying levels of degeneration was conducted (Fig. 2B). In nucleus pulposus cells with varying degrees of degeneration, the levels of HIF-1α and CITED2 were observed to be low, and their expression appeared to decrease as the degeneration worsened. Figure 2D displayed varying levels of degeneration in nucleus pulposus cells, where PPP1R15A and VEGFA were expressed. The expression of HIF-2α, CA9, and EGLN3 did not show significant differences in the study's database, and only a display of dimensionality reduction distribution was conducted.

### Gene Set Variation Analysis (GSVA) and pathway analysis to display hypoxia pathway gene set differences

**Fig. 3:**
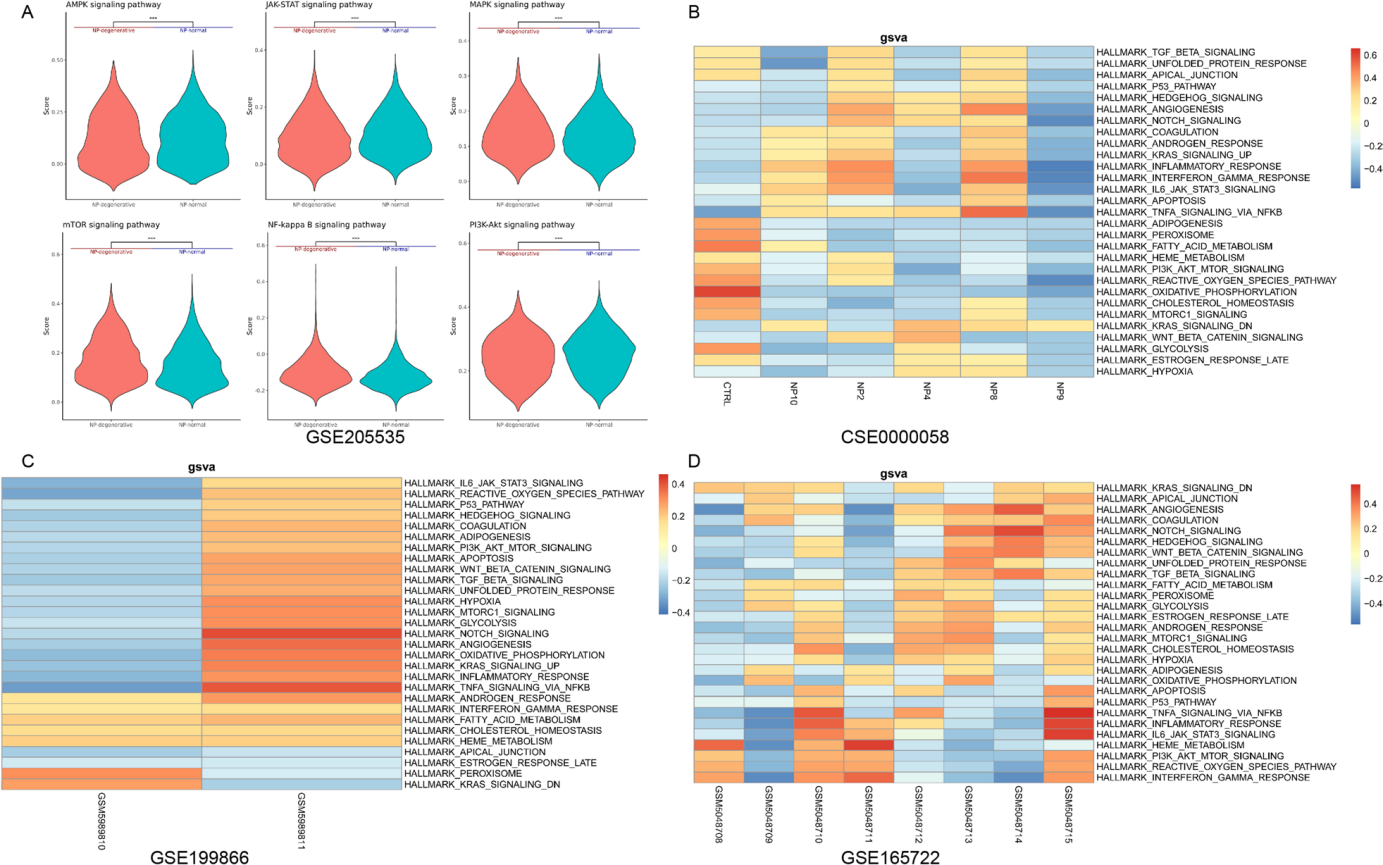
Comparisons of violin plots in the GSE205535 dataset showed variations in certain signaling pathways associated with oxygen metabolism between samples of healthy and degenerated nucleus pulposus in humans. (B.C.D) Heat map present GSVA scoring of Hallmark gene sets associated with hypoxia and angiogenesis in the CNP0002664 (CSE0000058), GSE199866, and GSE165722 database

Pathway enrichment analysis in the GSE205535 database revealed differences in some signaling pathways related to oxygen metabolism between human normal and degenerated nucleus pulposus samples, including AMPK, JAK-STAT, MAPK, mTOR, NF-kappa B, and PI3K-Akt (Fig. 3A).

Subsequently, we performed gene set variation analysis on three databases, CNP0002664 (CSE0000058), GSE199866, and GSE165722, and collected some variant gene sets associated with hypoxia and angiogenesis (Fig. 3 BCD). Certain patterns were consistently identified in degenerated nucleus pulposus samples, including Angiogenesis, Tnfα signalingvia NFKB, KRAS signaling, and Hypoxis, which were consistently enriched compared to normal nucleus pulposus samples. Although there is no clear pattern, the gene sets related to I3K-AKT-mTOR Signaling, Oxidative phosphorylation, Reactive oxygen species pathway, mTORC1 signaling, Glycolysis, and other hypoxia-angiogenesis show variations between human normal and degenerated nucleus pulposus samples.

### The production and analysis of PP20

**Fig. 4:**
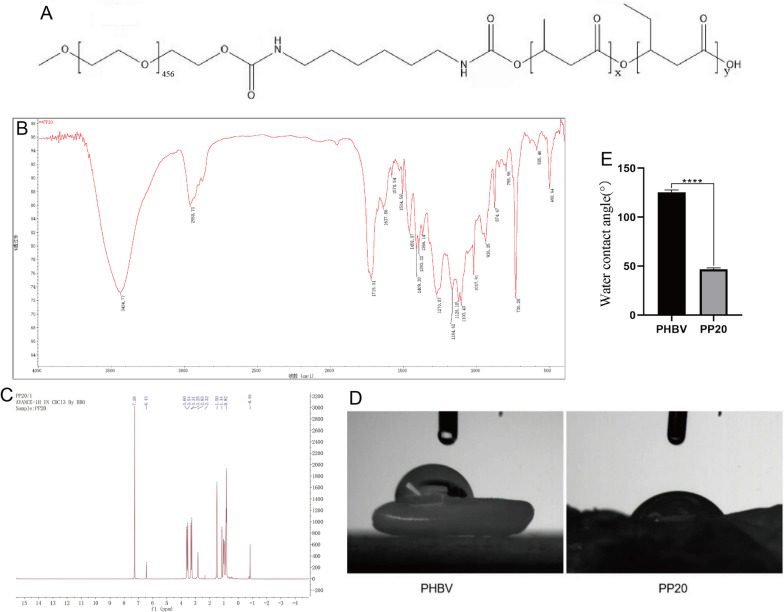
Properties of PP20 materials: **A** The chemical composition of PP20. **B** FT-IR spectra for the PP20. **C** The 1H NMR profiles of PP20. **D**, **E** Water contact angle of PHBV or PP20.Mean ± SD represents all the statistical data. (*n* = 3, ****p < 0.0001)

To surpass the constraints of PHBV, we chose to produce PP20 by utilizing PBHV and mPEG-20k as the main ingredients. The stretching and bending vibration of methylene in Fig. 4B is linked to the absorption peak around 2,958.73 and 1,409.30 cm−1 in the PP20 spectrum. Confirmation of the chemical composition of PP20 was achieved through 1H NMR analysis (Fig. 4C), wherein a comparison was made with the structural formula of PHBV and our previous discoveries [23]. This comparison verified the successful transplantation of mPEG20k onto PHBV [24].

To measure the water contact angle of liquid droplets on the scaffold surfaces, the sessile drop technique was employed. To determine the hydrophilicity of the scaffold surface, the water contact angle of PHBV (*n* = 3) and PP20 (*n* = 3) was measured using distilled water as an indicator (*n* = 3). Surface wettability is often assessed by measuring the water contact angle. The water contact angle measurements of the scaffolds, as depicted in Fig. 4 D,E, reveal that PHBV has a contact angle of 125° while PP20 has a contact angle of 46°. A surface that is hydrophilic can be identified by a water contact angle that is below 90°. Our findings suggest that PP20 may be more hydrophilic than PHBV, as it demonstrated a lower acute angle and may therefore facilitate greater cell adhesion.

### The release profile of PT2399 without any carrier and PT2399 encapsulated in PHBV and PP20 was examined in vitro

**Fig. 5:**
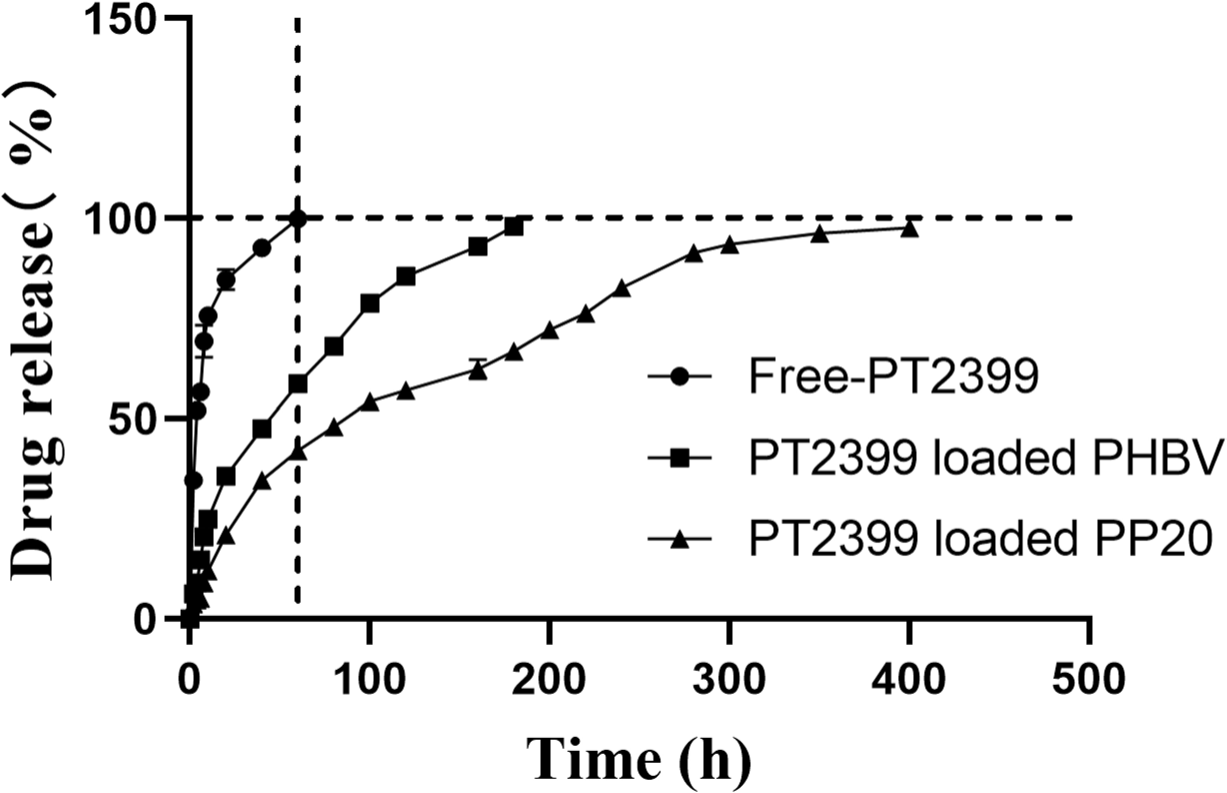
The release profile of PT2399 without any binding and PT2399 bound to PHBV and PP20 was examined in vitro

IDD is a chronic condition that necessitates extended therapy, with the expense of drug treatment being a major concern. Considerable alleviation of this problem can be achieved by improving the efficacy of medications. Multiple research studies have suggested that PHBV has the potential to enhance the effectiveness of medications. The drug release rate of PT2399 is examined by conducting tests when it is administered in two different forms: either freely or loaded onto PHBV or PP20. According to Fig. 5, our findings indicated that almost all of the PT2399 that was not bound was released within a span of 60 hours, whereas the PT2399 that was loaded onto PHBV demonstrated a release rate of around 68%. In contrast, PT2399 loaded onto PP20 showed a release rate of 48%. On the overall drug release curve, the release process of PT2399 loaded PP20 could be continuously released for more than 400 hours without sudden mass release, and the release efficiency of PT2399 could reach more than 97.6%. The results validate the capability of PHBV and PP20 in regulating the rates at which drugs are released. IDD necessitates extended treatment periods due to its persistent nature. Hence, PP20 might emerge as a more sophisticated alternative for IDD patients, providing enhanced regulation of gradual medication release. The findings indicate that employing PHBV or PP20 as drug transporters could improve the effectiveness of IDD treatment and potentially alleviate the burden of prolonged drug administration.

### The inhibition of HIF-2α regulates the gene expression changes in human nucleus pulposus cells following degeneration in the model

**Fig. 6:**
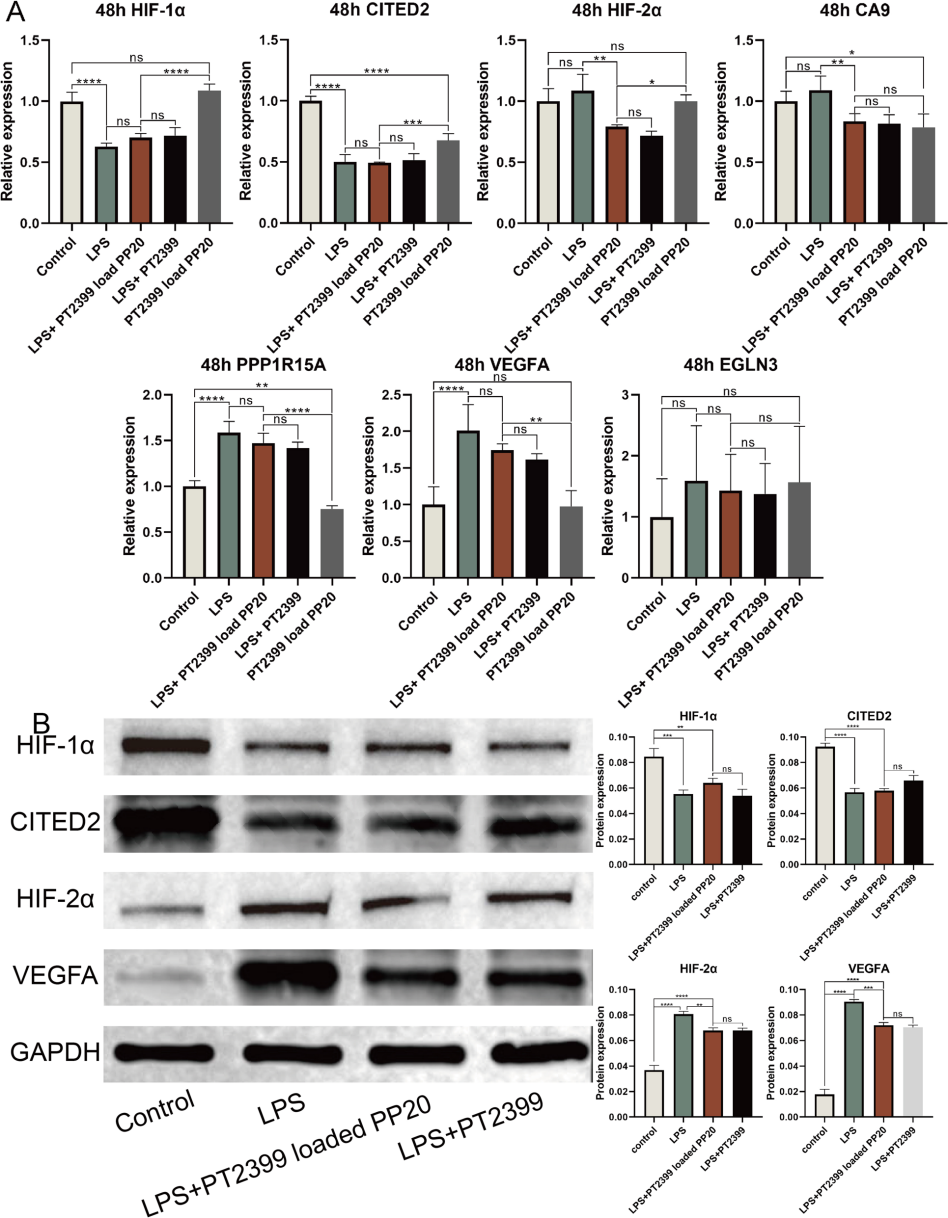
Gene expression changes in nucleus pulposus cells treated with each experimental group for 48 hours were detected using RT-qPCR. (B) Western bolt showed protein expression levels in different experimental groups.Mean ± SD represents all the statistical data. The significance levels are denoted as follows: (*p < 0.05, **p < 0.01, ***p < 0.001, ****p < 0.0001, ns indicates that the difference is not statistically significant)

Gene expression changes in nucleus pulposus cells treated with PBS (*n* = 4), LPS (*n* = 4), LPS + PT2399 loaded PP20 (*n* = 4), LPS + PT2399 (*n* = 4), and PT2399 loaded PP20 (*n* = 4) for 48 hours were detected using RT-qPCR (Fig. 6A). After LPS degeneration modeling, it was discovered that the expression of HIF-1α and CITED2 significantly decreased, whereas HIF-2α, CA9, PPP1R15A, VEGFA, and EGLN3 were up-regulated to different extents in comparison to the control group.Nevertheless, in the group subjected to LPS + PT2399 infused PP20, it was observed that the impact on HIF-1α and CITED2 was comparable to LPS alone, resulting in a decrease in expression levels. Compared with LPS alone, HIF-2α, CA9, PPP1R15A, VEGFA and EGLN3 decreased in LPS + free PT2399 and LPS + PT2399 loaded PP20. LPS + free PT2399 and LPS + PT2399 loaded PP20 had no significant difference in the effects on these parameters.

The western blot analysis was conducted to examine the alterations in the protein expression of nucleus pulposus cells treated with PBS (*n* = 3), LPS (*n* = 3), LPS + PT2399 loaded PP20 (*n* = 3), and LPS + free PT2399 (*n* = 3) for 48 hours (Fig. 6B). In comparison to the Control group, the levels of HIF-1α and CITED2 protein showed a tendency to decrease in LPS degenerated cells. However, there was no significant difference in the levels of these two proteins between LPS degenerated cells alone and LPS + free PT2399 or LPS + PT2399 loaded PP20 groups. Nevertheless, the levels of HIF-2α and VEGFA rose following LPS-induced degeneration of nucleus pulposus cells. Conversely, the expression decreased in the groups treated with LPS + free PT2399 and LPS + PT2399 loaded PP20. LPS + free PT2399 and LPS + PT2399 loaded PP20 had no significant difference in the effects on these parameters.

### PP20 loaded with PT2399 effectively inhibits angiogenesis in degenerated nucleus pulposus cells with a magnitude

**Fig. 7:**
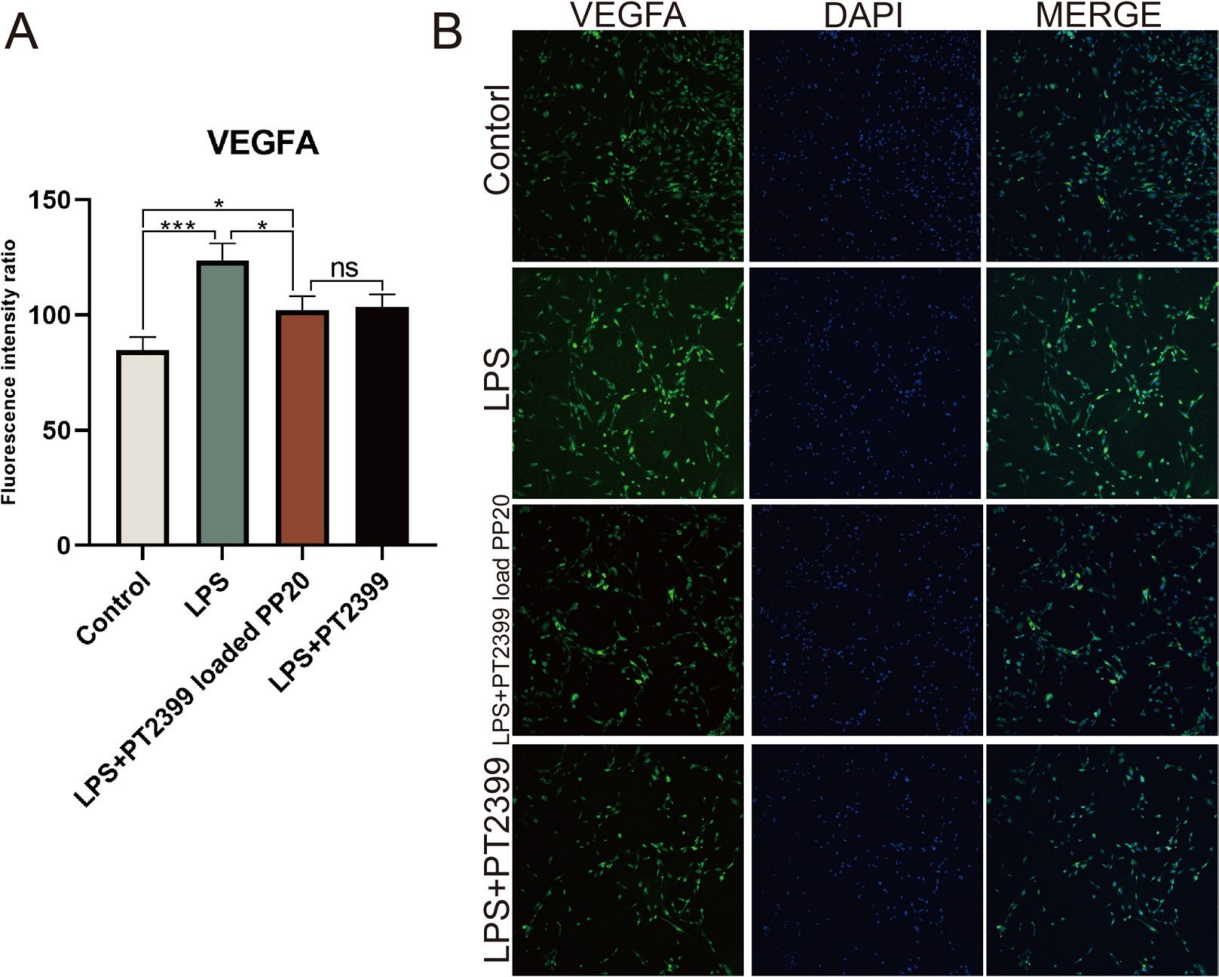
The immunofluorescence staining was used to determine the expression level of VEGFA in nucleus pulposus cells for each experimental group.Mean ± SD represents all the statistical data. (*n*=3, *p < 0.05, ***p < 0.001, ns no significant difference observed)

Immunofluorescence was used to measure the protein expression of VEGFA in nucleus pulposus cells treated with LPS (*n* = 3), LPS + PT2399 loaded PP20 (*n* = 3), and LPS + PT2399 (*n* = 3) for a duration of 48 hours (Fig. 7B). Compared to controls, VEGFA expression levels were observed to be increased in nucleus pulposus cells following LPS-induced degeneration modeling. In contrast, the experimental group that received PT2399 and PP20 loaded with PT2399 exhibited a decrease in VEGFA expression levels when compared to the LPS group (Fig. 7A).

### In the acupuncture experiment of rats intervertebral discs, the morphological changes were observed using Hematoxylin-eosin (HE) staining

**Fig. 8:**
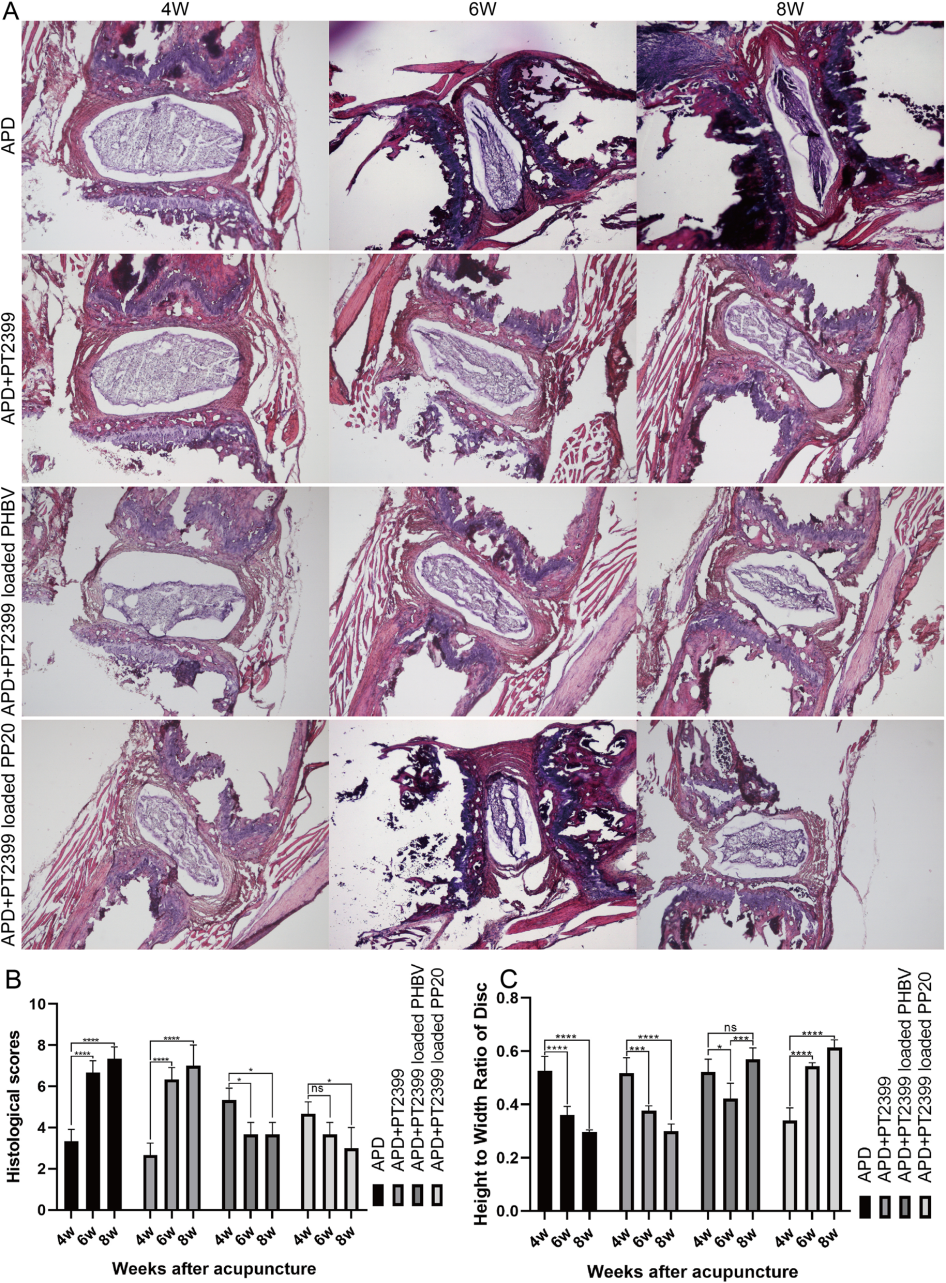
Histological scoring and height-to-width ratio statistics were performed after HE staining of APD or each experimental group injected rats. Mean ± SD represents all the statistical data. (*n*=3, **p < 0.01, ****p < 0.0001, ns no significant distinction)

The histological score and height-width ratio of the intervertebral disc were examined in rats at 4 weeks (*n* = 3), 6 weeks (*n* = 3), and 8 weeks (*n* = 3) after inducing degeneration and administering drugs via puncture injection (Fig. 8A). Histological scores increased more significantly and disc height-to-width ratio decreased significantly at 6 and 8 weeks compared to 4 weeks in the control (APD) and puncture injection PT2399 group. In the PT2399 loaded PHBV group and PT2399 loaded PP20 group (Fig. 8C), there was a slight decrease in histological scores and a slight increase in disc height-to-width ratio at 6 and 8 weeks compared to 4 weeks.

## Discussion

By utilizing bioinformatics analysis techniques and conducting in vitro validation experiments, we have acquired the subsequent findings: (1) HIF-1α and CITED2 [25] exhibited significant expression levels in healthy human nucleus pulposus cells, whereas HIF-2α (EPAS1), CA9 [26], PPP1R15A [27], VEGFA [26], and EGLN3 [28, 29] were observed to be upregulated in deteriorated nucleus pulposus cells. (2) HIF-2α, CA9, PPP1R15A, VEGFA, and EGLN3 tended to decrease to varying degrees in response to LPS + PT2399 loaded PP20. The hypoxic environment contributes to the degeneration of human nucleus pulposus tissue by regulating angiogenesis. By resisting angiogenesis during the degeneration of nucleus pulposus cells, PT2399 can effectively delay the degeneration of discs. The utilization of PT2399 load PP20 results in the gradual release of medications, consequently leading to the treatment of disc degeneration.

Earlier research has indicated that in healthy human nucleus pulposus tissues, there is an absence of blood vessels and a low oxygen environment, leading to dependence on anaerobic metabolism through glycolysis [5, 6]. Conversely, degenerated nucleus pulposus tissues experience the formation of blood vessels [7, 8]. The metabolism, function, and fate of disc nucleus pulposus cells are regulated by HIF-1α and HIF-2α, which are recognized for their crucial roles. The hypoxic ecology of nucleus pulposus cells and the mechanisms of energy metabolism, free radical mutation, and survival protein expression are regulated by these two factors that are sensitive to hypoxia [30]. In nucleus pulposus cells, HIF-1α controls both glycolysis, which is a form of anaerobic metabolism, and mitochondrial metabolism. On the other hand, the absence of HIF-2α does not affect disc development but does decrease fibrosis in nucleus pulposus tissue [11]. Prior research on the intervertebral disc has discovered that the expression of HIF-2α is notably increased in degenerated intervertebral discs of humans, potentially playing a role in regulating metabolism via the extracellular matrix [31]. In the experiment using a rat model of disc puncture degeneration, it was observed that the expression of HIF-2α was elevated in the group subjected to puncture [9]. The fundamental concepts of HIF-2α in maintaining stable levels in the intervertebral disc are constantly being investigated and clarified [32]. All of these findings indicate that HIF-2α could potentially contribute to the hastening of disc degeneration. Previous proposals suggest that the upregulation of gene programs related to angiogenesis, glycolysis, pH adaptation, and apoptosis is induced by HIF-1α and HIF-2α [33]. Previous research has shown that HIF-2α plays a role in controlling biological processes in different organ tissues including the gastrointestinal tract [34], bone [35], immune system [36], and blood vessels [37]. HIF-2α is linked to oxygen utilization, formation of new blood vessels, and various other physiological activities in different tissues [12–15]. Additionally, it has the potential to stimulate the growth of blood vessels and contribute to the processes of endochondral ossification in articular cartilage [38]. Based on the aforementioned studies, it can be inferred that the regulation of blood vessel formation by HIF-2α might play a crucial role in the progression of intervertebral disc degeneration. We performed enrichment analysis of GSVA and oxygen metabolism signaling pathways in two sample databases. These findings indicate alterations in the oxygen-deprived surroundings and regulation of blood vessel formation in the nucleus pulposus tissue of humans during degeneration. In order to confirm the significance of HIF-2α, we opted for PT2399, a specific inhibitor, which effectively suppressed the expression of HIF-2α in degenerated nucleus pulposus cells. Consequently, this led to a decrease in the expression of VEGFA, CA9, PPP1R15A, and EGLN3, indicating that these alterations could be attributed to the inhibition of HIF-2α. The findings indicate that HIF-2α might possess the capacity to control the balance of oxygen deficiency and the formation of new blood vessels in the nucleus pulposus.

Due to their ability to decompose naturally and their strong compatibility with living organisms, PHBV biopolymers have gained significant recognition and are extensively utilized in diverse sectors. The exceptional characteristics of this material, which include its flexibility and durability, render it a superb option for various medical uses, such as delivering medications, packaging medical supplies, stitching wounds, and creating cardiovascular stents. In order to improve the usage of PT2399, we incorporated it onto PHBV or PP20. After evaluating the water contact angle and drug release of these three delivery systems, we found that PP20 displayed greater hydrophilicity and outperformed the others. Hence, PP20 could be a more sophisticated alternative for administering patients with IDD. We explored the therapeutic efficacy of PHBV loaded with PT2399 and PP20 loaded with PT2399 in the APD rats model. The findings indicated that PP20 containing PT2399 demonstrated superior therapeutic efficacy in mitigating disc degeneration. To sum up, the PHBV biopolymer is a flexible substance with potential uses in the healthcare industry. The results of our research indicate that PHBV-based PP20 could be a more advantageous choice for treating IDD due to its exceptional hydrophilicity and superior efficacy in drug delivery.

The findings provide additional insights into the contrasting expression of hypoxia and angiogenic regulators in both healthy and degenerated nucleus pulposus cells in humans. They also explore the impact of HIF-2α on angiogenesis during the degeneration of nucleus pulposus tissue, comparing it to previous research. Moreover, they uncover alterations in the hypoxic conditions and angiogenesis that could potentially contribute to disc degeneration. The findings of this research shed light on the patterns of gene expression alterations in disc degeneration, addressing the previously unexplored impact of hypoxia-inducible factor on nucleus pulposus cells during degeneration. Moreover, it presents a novel potential treatment approach for disc degeneration by targeting HIF-2α inhibition, highlighting its importance and feasibility. The advancement of drug delivery systems based on PHBV-PEG20k (PP20) has the capability to transform IDD therapy by providing an eco-friendly, degradable, and compatible approach to drug delivery. Through additional examination, the PHBV-PEG20k (PP20) delivery system has the potential to offer a superior, secure, and convenient treatment alternative for individuals with IDD, ultimately resulting in enhanced health results and an improved standard of living.

## Materials and methods

### Data collection and preprocessing

We downloaded the datasets GSE205535, GSE199866, and GSE165722 from the Gene Expression Omnibus database. Database CNP0002664 was downloaded from China National GeneBank DataBase (CNGBdb). These databases contain single cell sequencing results from human normal and degenerated nucleus pulposus tissues. R software (version 4.2.0) and the Seurat package (version 4.0), developed by Satija Lab, were utilized for downstream clustering analysis, identification of differentially expressed genes, differential analysis, and identification of marker genes in single-cell data.

### Gene Set Variation Analysis (GSVA)

GSVA is an unsupervised analysis technique that evaluates the enrichment of gene sets on microarrays and transcriptomes, employing a non-parametric approach. The evaluation determines if various metabolic pathways are enriched in samples by transforming the gene expression matrix across samples into a gene set expression matrix across samples. pathways across different conditions or groups. a specific biological state is associated with a pathway [39].

GSVA (version 1.48.2) scoring of Hallmark gene sets, such as ANGIOGENESIS, TNFA_SIGNALING VIA NFKB, KRAS SIGNALLING, HYPOXIA was utilized in this study.

### Cell culture and drug intervention

Human normal nucleus pulposus cells (iCell-0028a, iCell Bioscience Inc, China) were utilized, while the cell culture medium was obtained from the iCell primary chondrocyte culture system (PriMed-iCELL-020, iCell Bioscience Inc, China). For the subsequent experiments, the nucleus pulposus cells were cultured in an incubator system set at a temperature of 37°C and a CO2 concentration of 5% after reaching the second passage.

The use of LPS (L2880, Sigma-Aldrich) can simulate the degeneration of nucleus pulposus cells by causing inflammation and cell degeneration. PT2399 selectively inhibits HIF-2α [16].

Slides were used to seed nucleus pulposus cells, which were then cultured in an incubator. After the cell density reached 50% -70%, PBS, LPS, LPS + PT2399, LPS + PT2399 loaded PP20 or PT2399 loaded PP20 were added to different groups. Cells were collected after 48 hours of drug exposure.

### Preparation and Characterization of PT2399 Loaded PHBV or PP20

To prepare PHBV-PEG20k (PP20), First, 8.0 g of mPEG-20k (Solarbio, China) was placed in a 50 mL round-bottom flask with a pumping valve, heated at 80 °C while vacuuming for 2 h, followed by nitrogen insufflation until cooled to room temperature. Once cooled, a round-bottom flask was used to transfer 40 mL of anhydrous dichloromethane, which was then dissolved by shaking for future use. The solution was subsequently transferred to a constant-pressure drip funnel and protected with nitrogen for later use. Transfer 100 μL of hexamethylene diisocyanate (HMDI) to a nitrogen-protected round-bottom flask and mix well with 20 mL of dry dichloromethane. mPEG-20k dissolved in dichloromethane was added to the HMDI solution while stirring at 800 rpm, with a flow rate of 1 mL/min. The reaction was carried out in the dark at room temperature for 6 hours to yield mPEG20k-NCO. In a 100 mL round-bottom flask equipped with a pumping valve, 13.3 grams of PHBV from Tianan Biologic Material Ltd. in China were heated at 80 °C under vacuum for 5 hours. After that, nitrogen was introduced until the temperature reached room temperature. Following the cooling process, a round-bottom flask was used to transfer 80 mL of anhydrous dichloromethane. The solution was dissolved by shaking and reserved for future use. Subsequently, the solution was transferred to a constant-pressure drip funnel and slowly added to the mPEG20k-NCO solution at a rate of 5 mL/min. This process took place at room temperature in the absence of light for a duration of 3 days. PHBV-PEG20k was obtained at the conclusion of the reaction by eliminating dichloromethane through rotary evaporation. It was then dissolved in 80 mL of tetrahydrofuran and the refined product was precipitated by gradually adding 1 L of distilled water while stirring. After filtration, the product was washed three times with distilled water and vacuum dried at 50 °C overnight.

Characterization of PP20, which is PHBV-PEG20k, is performed. The Perkin Elmer spectrophotometer was used to record the Fourier transform infrared spectra of PP20 (spectrum 2) via the solid-state KBr pellet technique, covering the 4,000–400 cm−1 range. The polymer's 1H-NMR was measured with a JEOL AL 500 FT-NMR Proton NMR spectrometer using FT-NMR. An internal reference was employed, utilizing tetramethylsilane.

To assess the water-loving properties of the scaffolds, we employed the sessile drop technique. This method entails using a contact angle analyzer (Surface Electro Optics, Phoenix 150, Korea) to measure the angle at which liquid droplets make contact with the surface of the scaffold. Each scaffold sample was gently covered with around 2 µL of distilled water, and the contact angles were documented. The immobile droplet technique is a commonly employed method for evaluating surface wetness, as it offers a numerical assessment of the relationship between the fluid and the surface. The degree of hydrophilicity or hydrophobicity of the scaffold surface can be determined by measuring the contact angle. A surface is more hydrophilic when the contact angle is smaller, and conversely, it is less hydrophilic when the contact angle is larger. Using a contact angle analyzer, like the Surface Electro Optics Phoenix 150, guarantees precise and uniform measurements. This method provides a reliable and efficient means of evaluating the hydrophilicity of scaffold surfaces, which is crucial for assessing their suitability for various biomedical applications.

The PT2399-loaded PHBV or PP20 was prepared using the double emulsion (W1/O/W2) technique. To summarize, 4.2 μg PT2399 was added gradually to 10 g of PHBV dissolved in chloroform using a homogenizer operating at 20000 rpm (SilentCrusher M, Heidolph, Germany) in order to create the initial emulsion (W1/O) whti a drug concentration of 1μmmol/L. This primary emulsion was then added to an aqueous phase (W2) containing PVA as an emulsifier and homogenized again. The resultant mixture was agitated until the liquid evaporated entirely and subsequently spun at a speed of 15000 revolutions per minute for a duration of 30 minutes using a centrifuge (MPW-350R, Poland). Afterwards, PHBV or PP20 loaded with PT2399 were rinsed three times using distilled water and then subjected to freeze-drying at a temperature of -50 °℃ for a duration of 24 hours in preparation for subsequent experiments.

### In vitro drug release behavior

The initial step involved creating a standard curve for PT2399 by utilizing PBS (pH = 7.4) as the solvent at the wavelength corresponding to the highest absorption peak (278 nm). PT2399-loaded PHBV, PT2399-loaded PP20 (100 mg) were each dissolved in PBS and placed in a dialysis bag. Next, the dialysis pouch was positioned inside a beaker filled with the identical pH PBS buffer. It was then subjected to horizontal vibrations in a temperature-controlled shaker (37 °C ± 0.5 °C) at a frequency of 50 vibrations per minute. At specific time intervals, the 5 ml of released liquid was substituted with equal quantities of new PBS. The solution's absorbance was measured at a wavelength of 278 nm, and the rate of drug release was calculated as follows.

$${\text{R}}(\mathrm{\%})=\frac{{\rho }_{n}V+{V}_{t{\sum }_{t}^{n-t}{\rho }_{t}}}{{M}_{D}}\times 100$$

The symbol R represents the total percentage of drug release, while n represents the number of samples taken. Indicates the amount of the medication in the nth release, measured in grams per liter. The release liquid's total volume is represented by V. The ith release is represented by the mass concentration (g/l) of the drug, while the volume of the release liquid at the ith sampling is denoted as Vi. MD refers to the mass of drug loaded (g).

### Detection of alterations in gene expression in nucleus pulposus cells following intervention with LPS and PT2399 loaded PP20 (RT-qPCR)

For a duration of 48 hours, human typical nucleus pulposus cells were exposed to LPS at a concentration of 10 µg/ml [40], PT2399 at a concentration of 1 ummol/L, and PT2399 loaded PP20 at a concentration of 1 ummol/L [41]. The expression levels of HIF-1α, CITED2, HIF-2α, CA9, PPP1R15A, VEGFA, and EGLN3 genes in nucleus pulposus cells from various experimental groups were detected using RT-qPCR. The verification of target differential genes in human normal and degenerated nucleus pulposus cell populations was done by analyzing the changes in target gene expression levels after each experimental group treatment. Additionally, the effects of drugs on HIF-2α and other indicators were also confirmed. The levels of mRNA were measured using the 2-ΔΔCt method, with GAPDH serving as the endogenous controls. The primer sequences were as follows: HIF-1α-F 5'-GCACAGTTACAGTATTCCAGCAGAC-3'; HIF-1α-R 5'-TTCATCAGTGGTGGCAGTGGTAG-3'. CITED2-F:5'-TCCTTGGTGATAGAAATGGGTTTGG-3'; CITED2-R:5'-CTCTGCTGGGCTGCTGTTTG-3’. HIF-2α-F:5'-TGATGTGGAAACGGATGAAGAACC-3'; HIF-2α-R:5'-TGGCAGCGGCAGATGTCTC-3'. VEGFA-F:5'-GGAGGAGGAAGAAGAGAAGGAAGAG-3'; VEGFA-R:5'-GCGGCTGGAGCACTGTCTG-3'. PPP1R15A-F:5'-ACAGAGGAAGAGGAAGATGAGGAAG-3'; PPP1R15A-R:5'-TGTAGCAGGAGTGGAAGAGGAAG-3'. CA9-F:5'-TGGCTGCTGGTGACATCCTAG-3'; CA9-R:5'-CTTCTGTGCTGCCTTCTCATCTG-3'. EGLN3-F:5'-TGGAGTACATCGTGCCCTGTC-3'. The sequences are as follows: EGLN3-R 5'-GCAGCGACCATCACCGTTG-3'; GAPDH-F 5'-GTCTCCTCTGACTTCAACAGCG-3'; and GAPDH-R 5'-ACCACCCTGTTGCTGTAGCCAA-3'.

### Analysis of western blot with a rating

The cells of the nucleus pulposus in every group were rinsed twice using PBS, then treated with 1% phenylmethanesulfonyl fluoride (PMSF) in RIPA lysis buffer (R0010, Solarbio) for a duration of 10 minutes. After that, they were subjected to centrifugation at 15,000×g for 10 minutes at a temperature of 4°C. The BCA protein assay kit (P0010, Beyotime) was used to measure protein concentrations. After that, the proteins were separated using 12.5% SDS-PAGE and then transferred to PVDF membranes (TM-PVDF-R-45, LABSELECT). Following a 90-minute incubation at room temperature with Western Blocking Solution (P0023B, Beyotime), the membranes were then subjected to overnight incubation at 4 °C with the primary antibodies HIF-1α (BF0593, Affinity, USA), CITED2 (DF2455, Affinity, USA), HIF-2α (DF2928, Affinity, USA), VEGFA (AF5131, Affinity, USA), and GAPDH (AF7021, Affinity, USA). Next, the membranes were exposed to secondary antibodies (LF102, EpiZyme, MA) for a duration of 1 hour at ambient temperature. Proteins of interest were identified following the established procedure. Blots were visualized using BeyoECL Plus (P0018FM, Beyotime, China). The quantification of the density of each band was performed using Image J version 1.8.0.

### Immunofluorescence stain

Prior to immunofluorescence, cultured nucleus pulposus cells were treated with 4% PFA and fixed for a duration of 20 minutes. After treating the cells with 0.5% Triton X-100 for 20 minutes to make them permeable, they were then subjected to a 10-minute blocking step at room temperature using 5% BSA. After incubating overnight with primary antibodies targeting VEGFA (AF5131, Affinity, USA) at a temperature of 4 ° C, the samples were then exposed to secondary antibodies (LF102, EpiZyme, MA) for a duration of 1 hour at ambient temperature.BY-1002) to mount the slides and prevent fading of the DAPI stain. P0131) for installation. Acquisition of images was performed with a confocal microscope, specifically the Zeiss LSM 880.

### Rats were subjected to experiments, followed by histological staining and grading using a scoring system

Prior to commencing animal experiments, this study received approval from the Ethics Committee of the Affiliated Hospital at Guangdong Medical University.

This study utilized 36 Sprague–Dawley (SD, YC-SD004) rats. The 36 rats used in the experiment were divided into four groups randomly: Acupuncture-induced pathological degeneration group (APD), Acupuncture with PT2399 injection (APD+PT2399), Acupuncture with PT2399-loaded PHBV injection (APD+PT2399-loaded PHBV), and Acupuncture with PT2399-loaded PP20 injection (APD+PT2399-loaded PP20). Each experimental group consisted of nine rats subjected to different experimental methods. A 1 milliliter syringe (HSZSQ-1ML-01, KERONG, CHINA) having a 0.45 mm diameter was employed for administering a dose of PT2399, equivalent to 10 nanomoles per kilogram, into the middle disc of every rat. Each rat was kept in separate enclosures, provided with unrestricted access to water and food, and their well-being was monitored on a daily basis. The Ethics Committee of Affiliated Hospital of Guangdong Medical University approved all animal experiments.

Tissue staining was performed on the intervertebral discs and nearby tissues at the surgical site of two groups of rats at 4 (*n* = 3), 6 (*n* = 3), and 8 (*n* = 3) weeks after degeneration or drug injection following puncture.

The tissues were fixed in a solution containing 10% neutral-buffered formalin and 10% cetylpyridinium chloride. Following decalcification using Cal-Ex II Fixative/Decalcifier (Fisher Scientific, Pittsburgh, PA, USA), the specimens were then immersed in paraffin and sliced into sections measuring 6 m in thickness.

Hematoxylin and eosin (H&E) were used to stain the cellular components of the sections, while safranin-O was used to stain the proteoglycans. After deparaffinization and rehydration, the sections were stained using the H&E staining kit (Solarbio, Beijing, China), safranin-O cartilage staining kit (Solarbio), and alcian blue staining kit (Solarbio) as per the provided instructions. The pictures were taken with a Leica light microscope from Wetzlar, Germany, at magnifications of 50× and 40×.

Assessing the level of deterioration involved employing a revised grading system (Supplementary Table 1), which drew inspiration from both rabbit and human grading systems. The grading scores for AF and NP varied between 1 and 4. To assess the extent of degeneration, the scores of AF and NP were combined.

### Reagent

LPS (L2880, Sigma-Aldrich), mPEG-20k (Solarbio, China), PHBV (Tianan Biologic Material Ltd, in China), PT2399 (HY-108697, MCE, USA), PBS (HY-K3005, MCE, USA), RIPA lysis buffer (R0010, Solarbio), BCA protein assay kit (P0010, Beyotime), PVDF membranes (TM-PVDF-R-45, LABSELECT), Western Blocking Solution (P0023B, Beyotime), BeyoECL Plus (P0018FM, Beyotime, China), Cal-Ex II Fixative/Decalcifier (Fisher Scientific, Pittsburgh, PA, USA), H&E/safranin-O cartilage/alcian blue staining kit (Solarbio, Beijing, China). The RT-qPCR primer sequences ( Sangon, China), The WB and Immunofluorescence primary antibodies (Affinity, USA), The WB and Immunofluorescence secondary antibodies (LF102, EpiZyme, MA).

#### Statistical analysis

All statistical analyses and figures in the online database were generated using R software (version 4.2.0). All data in the experiments were analyzed and histograms were generated using GraphPad Prism software (version 8.0).

## Conclusions

In summary, this study pioneered the development of a promising delivery system, PP20 loaded with PT2399, with sustained release drug effects by a method. PT2399 load PP20 effectively regulates the hypoxic environment and angiogenesis in nucleus pulposus cells, thereby treating disc degeneration, as evidenced by the differential expression of HIF-2α and other genes in human normal and degenerated nucleus pulposus tissues. Hence, this research offers new indicator genes for the deterioration of discs and expands the comprehension of the mechanism behind disc degeneration. Targeting HIF-2α could potentially offer a novel approach to address disc degeneration, thereby establishing a theoretical foundation for future therapeutic interventions. At the same time, this study took the lead in targeting HIF-2α to explore a more promising possibility option for PT2399 loading PP20 in more comprehensive clinical treatments for disc degeneration.

### Supplementary Information

**Additional file 1: Supplementary Table 1.** Modified Mouse Intervertebral Disc Histology Score.

## Data Availability

The raw data of transcriptome sequencing are available at GEO DateSets of NCBI (NO.GSE205535, GSE199866 and GSE165722) and Transcriptome or Gene expression of CNGBdb (NO.CNP0002664).
